# Supplementary material for: Adipose stem cell niche reprograms the colorectal cancer stem cell metastatic machinery
Source: Nat Commun. 2021 Aug 18;12:5006. doi: 10.1038/s41467-021-25333-9 (PMC8373975; doi:10.1038/s41467-021-25333-9)
Supplement: Supplementary file 1 — Supplementary Information [file 41467_2021_25333_MOESM1_ESM.pdf]

a

Variables	Univariate analysis			Multivariate analysis		
	Patients #	HR (95% CI for HR)	p value	Patients #	HR (95% CI for HR)	p value
BMI (>=30 vs >18.5 & <30)	511	1.8 (1.3-2.4)	0.00012	341	1.82 (1.3 - 2.6)	0.00094
Stage (3-4 vs 1-2)	488	2.3 (1.7-3.1)	3.10E-07		3 (2.1 - 4.4)	8.50E-09
Curative Surgery (1 vs 0)	360	0.19 (0.1-0.36)	5.20E-07		0.109 (0.055 - 0.21)	1.80E-10
Adjuvant Regimen (1 vs 0)	356	1.3 (0.95-1.8)	0.097		0.887 (0.62 - 1.3)	0.51

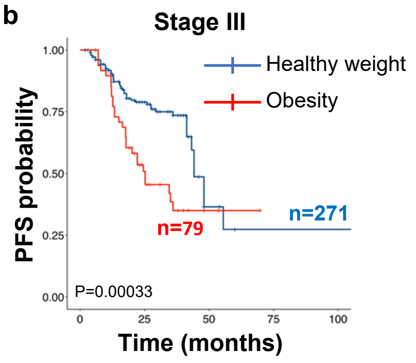

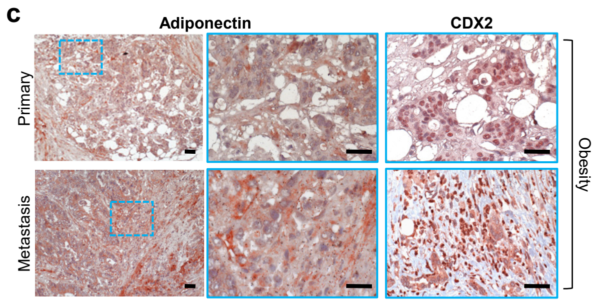

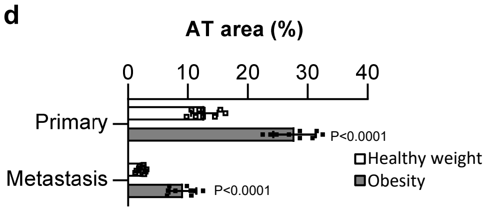

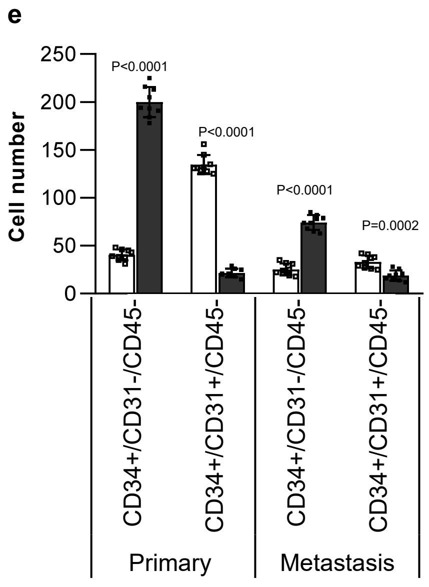

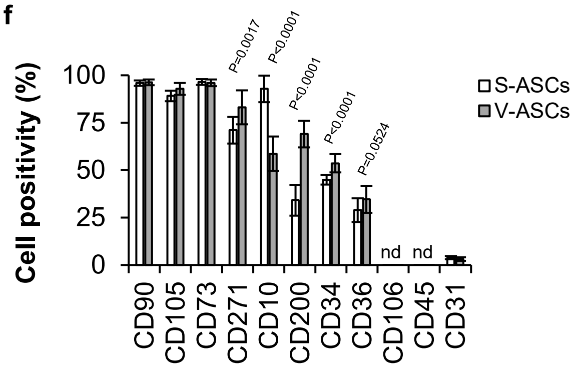

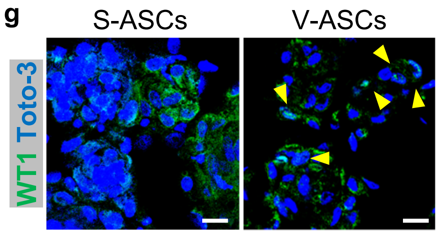

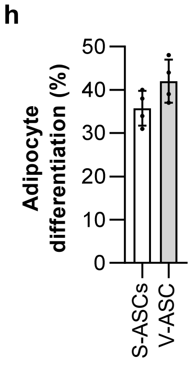

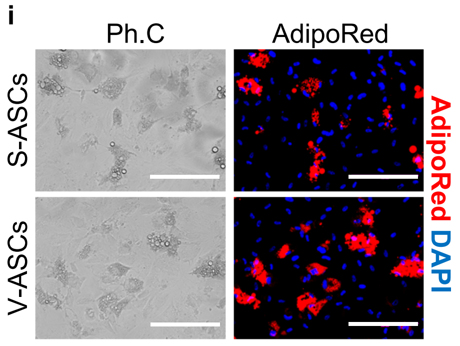

Di Franco et al. Supplementary Figure 1

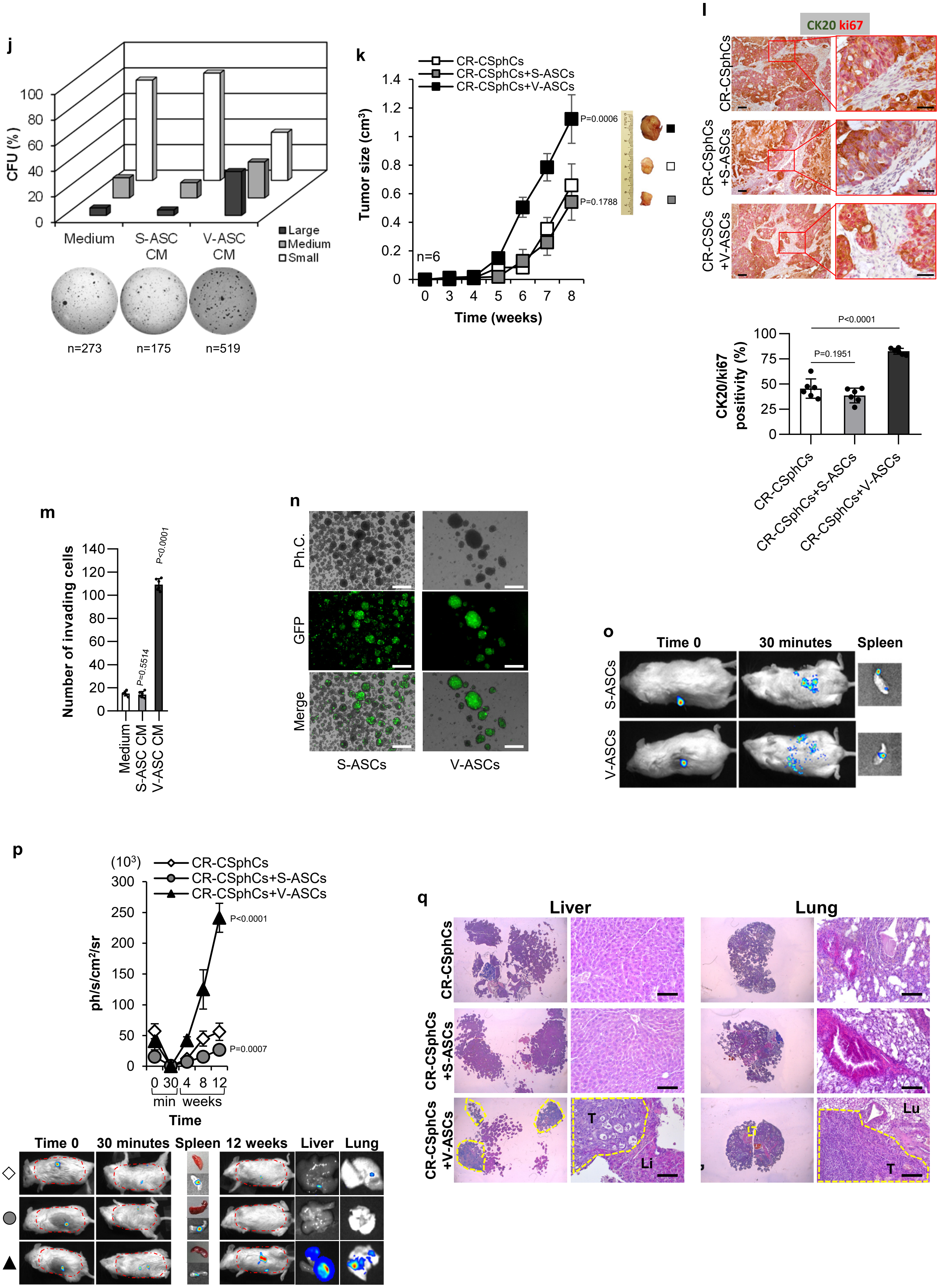

Di Franco et al. Supplementary Figure 1

Supplementary Fig. 1. V-ASCs sustain the metastatic potential of CR-CSphCs.

a, Univariate and multivariate analysis of progression free survival (PFS) according to regression Cox model in CRC patients. Statistical significance was calculated using the Wald test. **b**, Kaplan-Meier PFS curves in stage III CRC patients, based on BMI status. Healthy weight indicates 18,5<BMI<30, and obesity BMI>30. Statistical significance was calculated using the log-rank (Mantel–Cox) test. **c**, Immunohistochemical analysis of adiponectin and CDX2 on primary and liver metastasis tissue specimens from CRC patients with obesity. One representative of 9 independent experiments is shown. Scale bars, 100 µm. **d**, Percentage of adipose tissue (AT) area based on adiponectin positivity, evaluated in primary tumor and liver metastasis as in (c). **e**, Number of $CD34^+/CD31^-/CD45^-$ and $CD34^+/CD31^+/CD45^-$ cells on primary and liver metastasis CRC from patients with healthy weight or affected by obesity. For (d, e) data are mean ± standard error of 9 independent experiments. **f**, Mesenchymal stem cell and endothelial (CD31, CD34) marker expression in ASCs isolated from subcutaneous (S-ASCs; n= 13) and visceral (V-ASCs; n= 10) adipose tissue. **g**, Immunofluorescence analysis of WT1 expression in S-ASCs or V-ASCs. Yellow arrow heads indicate ASCs with nuclear localization of WT1. Scale bars, 40 µm. Data are representative of experiments performed in 12 S-ASCs and 10 V-ASCs. **h**, Adipocyte differentiation of S-ASCs or V-ASCs. Data are mean ± S.D. of 4 independent experiments using 3 different S-ASC and V-ASC cultures. **i**, Phase contrast and fluorescence analysis of lipid droplets content in cells as indicated. Scale bars, 100 µm. One representative of 4 independent experiments is shown. **j**, Colony forming assay of CR-CSphCs treated as indicated. *n* represents number of colonies. **k,** Size of subcutaneous tumor xenografts at the indicated time points generated by injection of CR-CSphCs alone or together with S-ASC or V-ASCs. **l,** Immunohistochemical analysis of CK20 (brown) and ki67 (red) of tumor xenografts generated by subcutaneous injection of cells as in (k) *(upper panel)*. Percentage of $ki67^+/CK20^+$ cells in tumor xenografts. **m,** Invasion assay of CR-CSphCs treated as indicated for 48 hours *(lower panel)*. **n,** GFP expression of transduced LUC-GFP S-ASCs and V-ASCs. Scale bars, 500 µm. One representative of 3 independent experiments is shown. **o**, Whole body *in vivo* imaging analysis 30 minutes after intrasplenic injection of ASCs transduced with LUC-GFP. **p**, Kinetics of *in vivo* whole-body imaging analysis of metastasis formation following intrasplenic injection of LUC-GFP transduced CR-CSphCs alone and together with S-ASCs or V-ASCs at the indicted time points. Bioluminescence signal of isolated organs was detected after splenectomy at the indicated time. Red dotted line indicates the area of photons quantification. For (j-m, and p) data are mean ± SD of 6 independent experiments using 4 different CR-CSphC lines (#1, #8, #9 and #21). **q**, H&E analysis of liver and lung metastases derived from tumor xenografts generated by intrasplenic injection of indicated cells. Scale bars, 200 µm. Li: liver; Lu: lungs; T: tumor. For (o-q) one representative of 6 independent experiments is shown. Statistical significance between 2 groups was determined by unpaired Student's t-test (2-tailed).

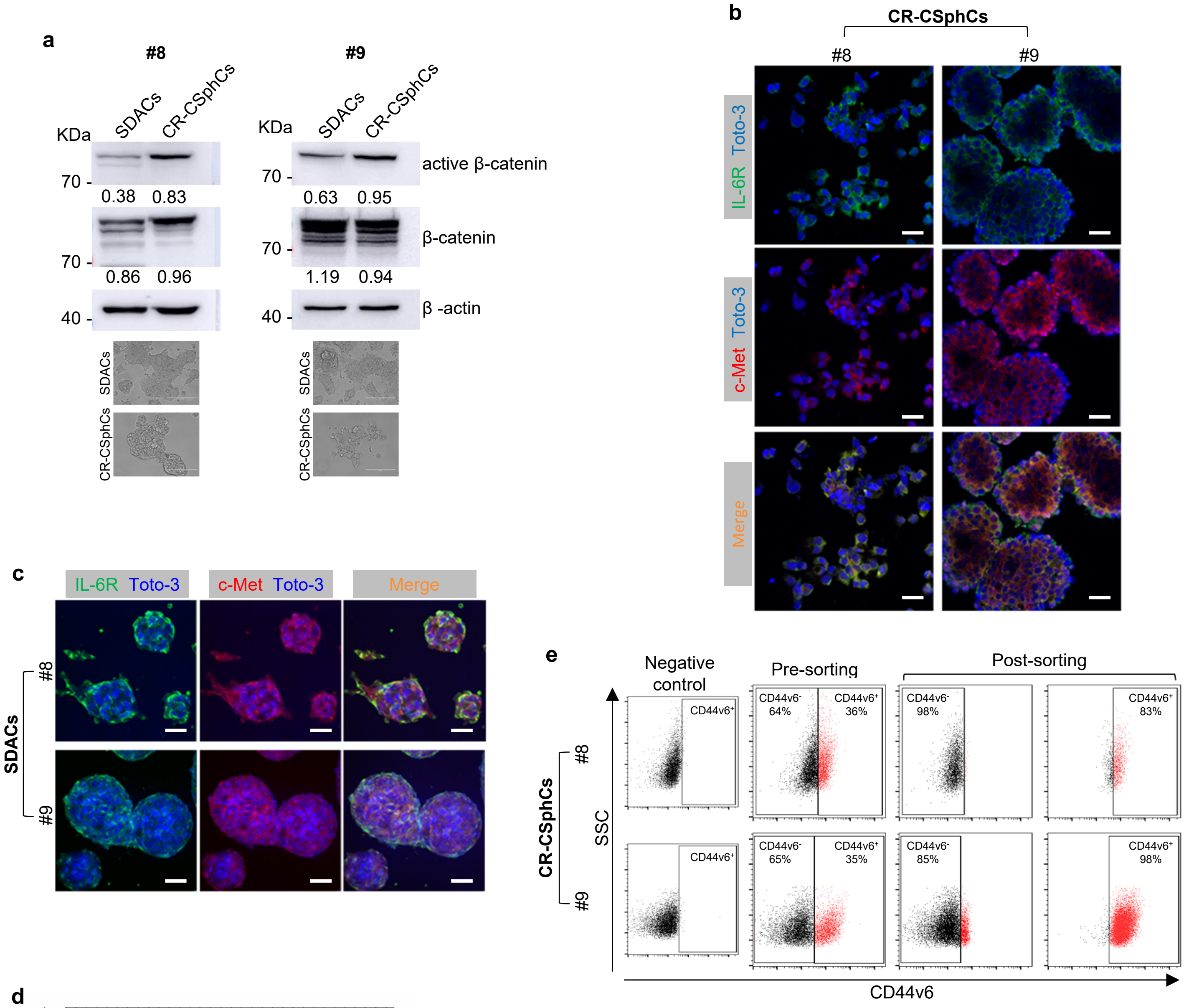

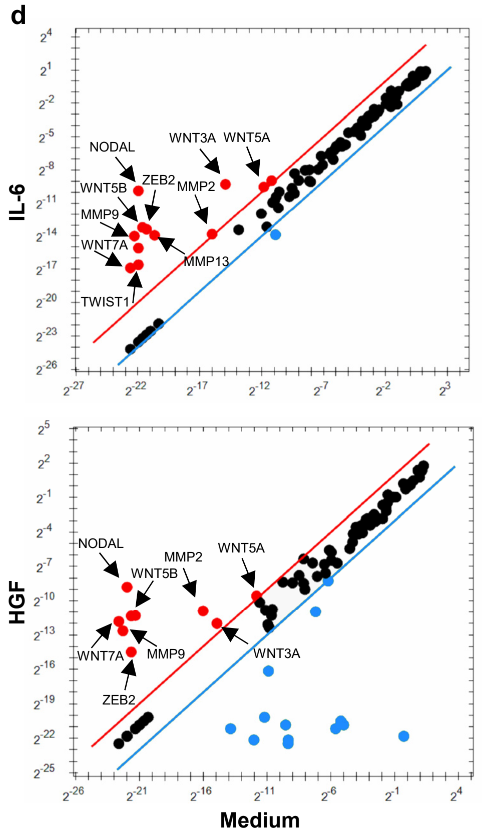

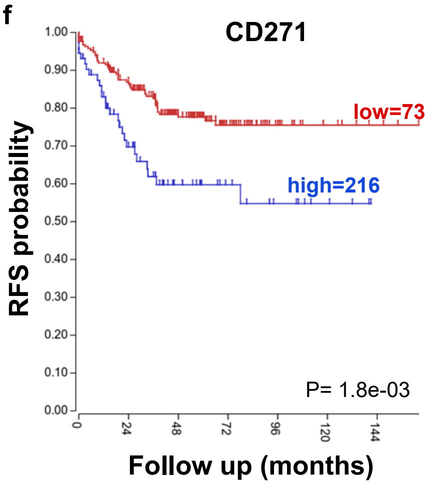

g

Variables	Univariate analysis		Multivariate analysis	
	HR (95% CI)	p value	HR (95% CI)	p value
**Gender** (F vs M)	1.4 (0.87-2.4)	0.16	1,26 (0,69 - 2,32)	0.44
**Age** (>60 vs <60)	0.87 (0.49-1.5)	0.62	0,58 (0,30 - 1,15)	0.12
**Stage** (III-IV vs I-II)	1.3 (0.81-2.2)	0.26	1.11 (0,60 - 2,05)	0.72
**KRAS** (mut vs wt)	1.2 (0.68-2.2)	0.5	1,31 (0,69- 2,50)	0.4
**Cimp** (neg vs pos)	1.2 (0.67-2.3)	0.51	1.33 (0,71 - 2,48)	0.37
**CD271** (high vs low)	1.8 (0.95-3.4)	0.073	2,38 (1,07- 5,29)	0.032

Di Franco et al. Supplementary Figure 2

Supplementary Fig. 2. CRC spheres and their derived adherent cells express IL-6R and c-Met.
a, Immunoblot analysis of non phospho active and total β-catenin in CMS2 CR-CSphCs (#8 and #9) and sphere-derived adherent cells (SDACs). β-actin was used as loading control *(upper panel)*. Samples were run on the same gel and images were cropped only for the purpose of this figure. Source data are provided as a Source Data file. Representative phase contrast analysis of cell morphology in CMS2 CR-CSphCs (#8 and #9) and sphere-derived adherent cells (SDACs). Scale bars, 200 μm *(lower panel)*. **b**, Immunofluorescence analysis of IL-6R and c-Met in CMS2 CR-CSphCs (CSphC #8, #9). Nuclei were counterstained with Toto-3. **c**, Immunofluorescence analysis of IL-6R and c-Met in CMS2 SDACs (#8, #9). For (a-c) data are representative of 4 independent experiments. For (b-c) scale bar, 40 μm. **d**, Stemness-related genes in CMS2 CR-CSphCs (#8, #9) treated with vehicle (Medium), IL-6 or HGF for 48 hours. Black arrows indicate up- (red) and down- (blue) regulated genes. *GAPDH* and *HPRT1* were used as housekeeping control genes. **e**, Flow cytometry profiles of CD44v6 in $CD44v6^-$ and $CD44v6^+$ sorted CR-CSphCs (CSphC #8, #9). Gating strategy to sort $CD44v6^-$ and $CD44v6^+$ CR-CSphCs used on the *in vitro* assays presented on Fig. 2j-m. **f**, Relapse-free survival (RFS) rate of CMS2 CRC patients according to CD271 expression levels. Statistical significance was calculated using the log-rank (Mantel–Cox) test. **g,** Univariate and multivariate analysis of relapse free survival (RFS) according to regression Cox model in CRC patients as in (f). Statistical significance was calculated using the Wald test.

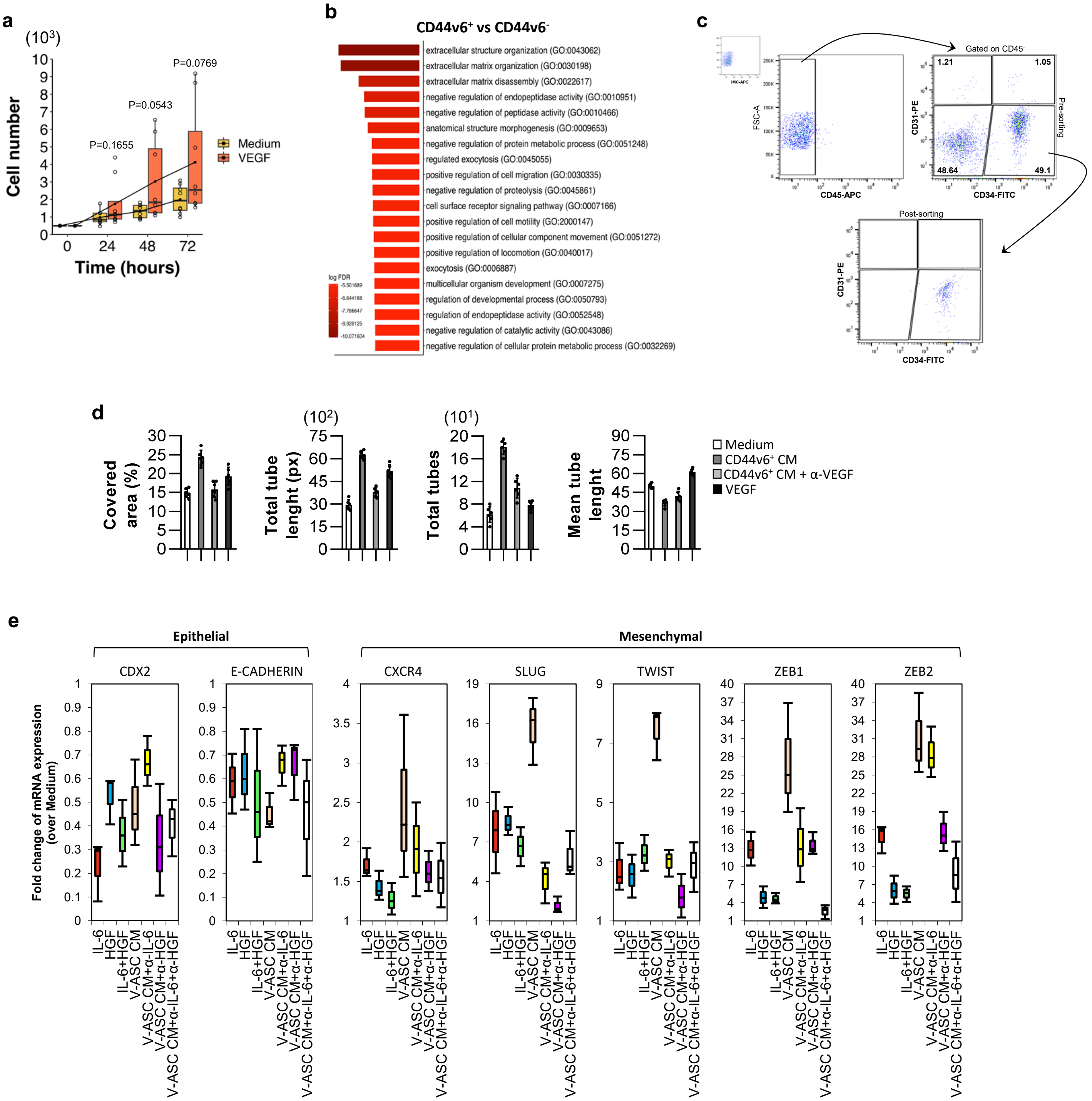

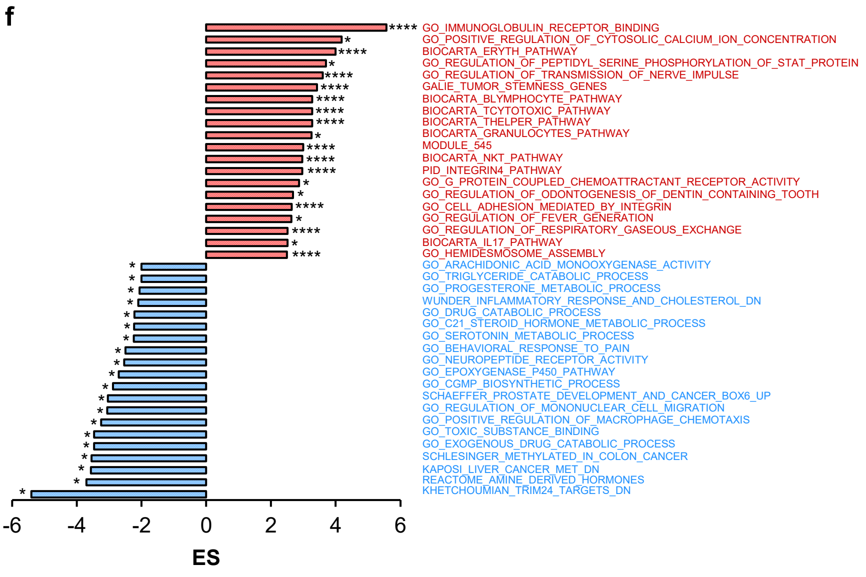

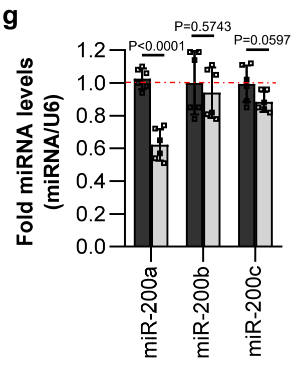

Di Franco et al. Supplementary Figure 3

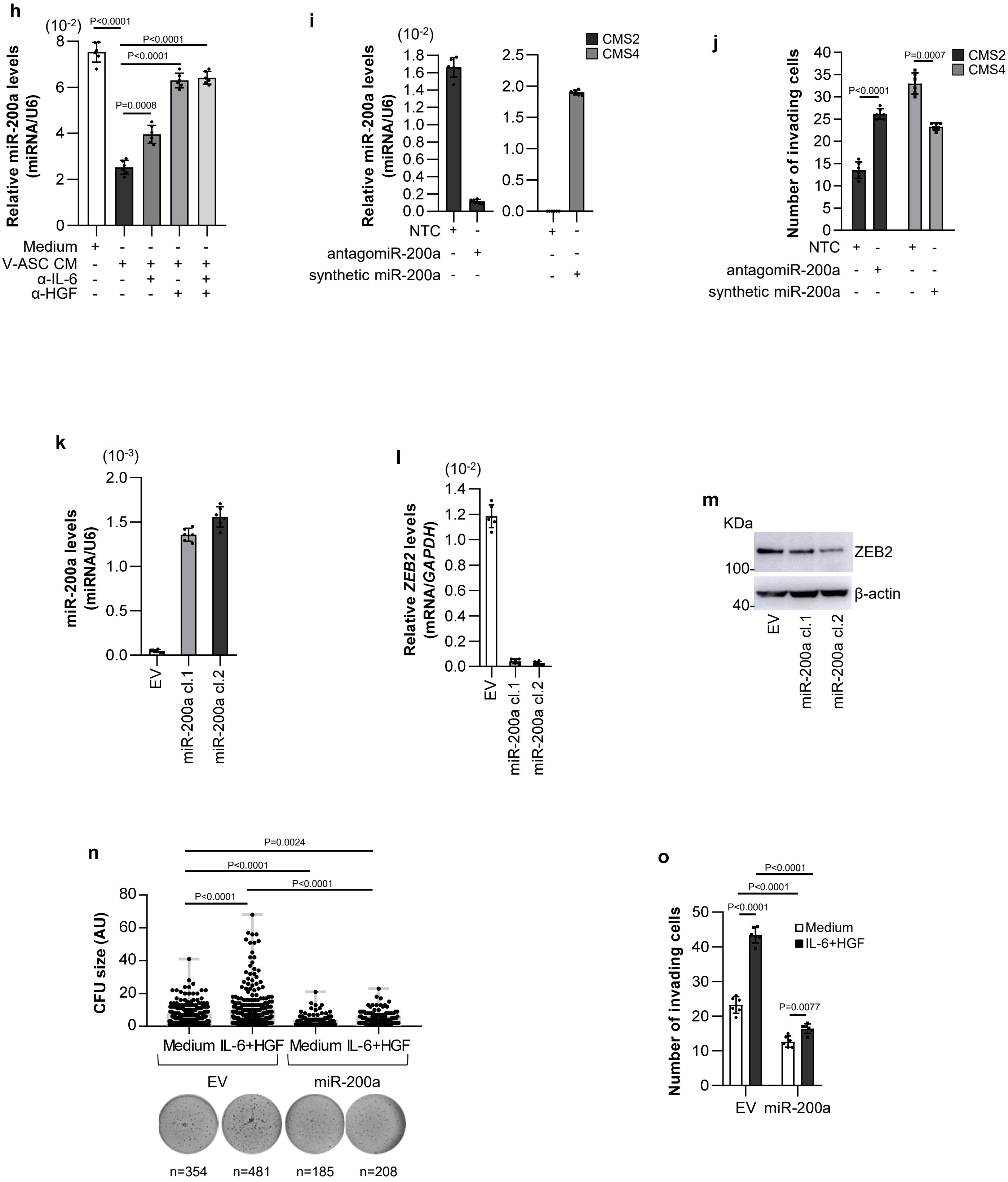
h
(10-2)
Relative miR-200a levels (miRNA/U6)
P<0.0001
P<0.0001
P<0.0001
P=0.0008
Medium + - - - -
V-ASC CM - + + + +
α-IL-6 - - + - +
α-HGF - - - + +
i
(10-2)
Relative miR-200a levels (miRNA/U6)
CMS2
CMS4
NTC + - + -
antagomiR-200a - + - -
synthetic miR-200a - - - +
j
Number of invading cells
P<0.0001
P=0.0007
CMS2
CMS4
NTC + - + -
antagomiR-200a - + - -
synthetic miR-200a - - - +
k
(10-3)
miR-200a levels (miRNA/U6)
EV
miR-200a cl.1
miR-200a cl.2
l
(10-2)
Relative ZEB2 levels (mRNA/GAPDH)
EV
miR-200a cl.1
miR-200a cl.2
m
KDa
ZEB2
100-
β-actin
40-
EV
miR-200a cl.1
miR-200a cl.2
n
P=0.0024
P<0.0001
P<0.0001
P<0.0001
CFU size (AU)
Medium IL-6+HGF Medium IL-6+HGF
EV
miR-200a
n=354 n=481 n=185 n=208
o
P<0.0001
P<0.0001
P<0.0001
P=0.0077
Number of invading cells
Medium
IL-6+HGF
EV miR-200a

Supplementary Fig. 3. Adipokines down-regulate miR-200a improving clonogenic and invasive activity of CR-CSphCs

a, Box and whiskers plot showing cell growth of 8 different ASCs treated as indicated. Box and whiskers show min-to-max values, with line indicating the median value, and the connecting lines indicating the mean values of 8 independent experiments. Statistical significance was calculated using two-tailed paired nonparametric Mann–Whitney test. **b**, Top twenty significantly enriched gene sets from PANTHER (GO biological process annotation) (FDR p-value ≤ 0.05) identified through the analysis of differentially expressed genes in CR-CSphCs (#1, #8, #9, #21). **c**, Gating strategy to sort $CD31^-/CD34^+/CD45^-$ ASCs used on the *in vitro* endothelial-differentiation assay presented on Fig. 3d. **d**, Tube formation assay analysis of Huvec cells exposed to vehicle (Medium), CM derived from $CD44v6^+$ CR-CSCs (#1, #8, #9, #21), in presence or absence of VEGF neutralizing antibody, or VEGF for 16 hours. **e**, Boxplot of epithelial and markers expression in CMS2 cells treated as indicated for 48 hours. Box and whiskers show min-to-max values, with line indicating the mean value. **f**, Up- (red) and down- (blue) regulated signaling pathways, computed by GSEA, in CMS2 CR-CSphCs (#8, #9) treated with V-ASC conditioned medium. **g**, miR-200 family members expression in CMS2 CR-CSphCs (#8, #9) following treatment with V-ASC CM. **h**, miR-200a expression levels in CMS2 CR-CSphCs (#8, #9) treated as indicated. **i**, miR-200a expression levels in CMS2 (#8, #9) and CMS4 CR-CSphCs (#1, #21) transfected with antagomiR-200a or synthetic miR-200a, respectively. **j**, Invasion assay of CMS2 (#8, #9) and CMS4 (#1, #21) CR-CSphCs as indicated. **k**, miR-200a expression in CR-CSphCs (#1, #21) transduced with EV or miR-200a. **l**, *ZEB2* expression levels in CR-CSphCs (#1, #21) transduced as indicated. **m**, Immunoblot analysis of ZEB2 in CMS4 CR-CSphCs (#1, #21) transduced as in (k). β-actin was used as loading control. One representative of 6 independent experiments is shown. Samples were run on the same gel and images were cropped only for the purpose of this figure. Source data are provided as a Source Data file. **n**, Colony forming assay of CMS2 CR-CSphCs transduced with empty vector (EV) or cl.2 miR-200a and treated as indicated, at 21 days. Data represent mean ± S.D. of colony size performed in 4 independent experiments using 2 different CR-CSphC lines (#8, #9). *n* represents the number of colonies. Statistical significance was calculated using two-tailed nonparametric Mann–Whitney test. **o**, Number of invading CMS2 (#8, #9) and CMS4 (#1, #21) CR-CSphCs transduced as in (n) and treated as indicated. For (d, g-l and o) data are shown as mean ± S.D. of 6 independent experiments. Statistical significance between 2 groups was determined by unpaired Student's t-test (2-tailed). **** $p \leq 0.0001$; * $p \leq 0.05$.

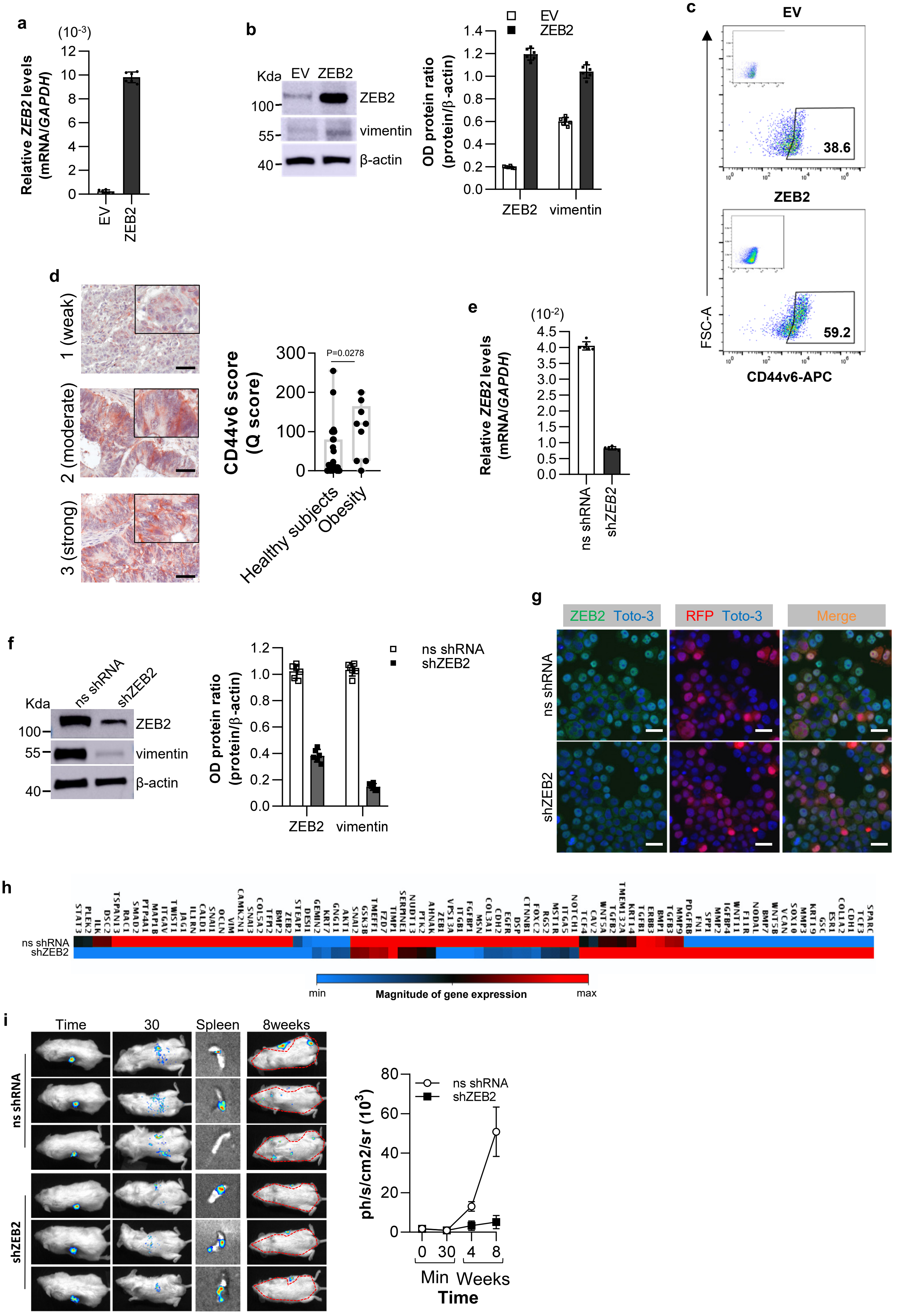

Di Franco et al. Supplementary Figure 4

Supplementary Fig. 4. ZEB2 is crucial for CRC metastasis formation

a, ZEB2 expression in CMS2 CR-CSphCs (CSphC #8, #9) transduced with EV or ZEB2. *GAPDH* was used as housekeeping control gene. **b**, Immunoblot analysis of ZEB2 and vimentin expression in CR-CSphCs (CSphC #8, #9) transduced as indicated. β-actin was used as loading control. Samples were run on the same gel and images were cropped only for the purpose of this figure. Source data are provided as a Source Data file. **c,** Representative flow cytometry analysis of CD44v6 on CR-CSphCs transduced with empty vector (EV) or ZEB2. **d,** Immunohistochemical analysis of CD44v6 on CRC tissue with weak (1), moderate (2), and strong (3) staining intensity. Scale bars represent 100 µm. CD44v6 score (Q score = percentage of positive cells (P) x intensity (I)) in) CRC patients with healthy weight (n=28) and obesity (n=9. Box and whiskers show min-to-max values, with line indicating the mean value. **e**, *ZEB2* expression levels in CMS4 CR-CSphCs (CSphC #1, #21) transduced with inducible nonsilencing (ns) shRNA control, or ZEB2 shRNA, following 72 hours of exposure to doxycycline. *GAPDH* was used as housekeeping control gene. **f**, Immunoblot analysis of ZEB2 and vimentin in cells transduced as in (c). β-actin was used as loading control. Samples were run on the same gel and images were cropped only for the purpose of this figure. Source data are provided as a Source Data file. For (a-b and e-f) data represent mean ± S.D. of 6 independent experiments. **g**, Immunofluorescence analysis of ZEB2 in RFP tagged CR-CSphCs (CSphC #1, #21) transduced as indicated. Scale bars, 40 µm. One representative of 6 independent experiments is shown. **h**, Heatmap of EMT-related genes in CMS4 CR-CSphCs (CSphC #1, #21) transduced as indicated. Data are mean of 3 independent experiments. **i**, Whole body *in vivo* imaging analysis of mice (n=6) intrasplenically injected with CMS4 CR-CSphCs (CSphC #1, #21) transduced as indicated, at 8 weeks after splenectomy. Spleen was removed 30 minutes after cell injection. Red dotted line indicates the area of photons quantification. *(right panel)* Luciferase signal measured as photons per second that leave a square centimeter of tissue and radiate into a solid angle of one steradian (ph/s/cm^2/sr). One representative of 6 independent experiments is shown. Statistical significance between 2 groups was determined by unpaired Student's t-test (2-tailed).

a

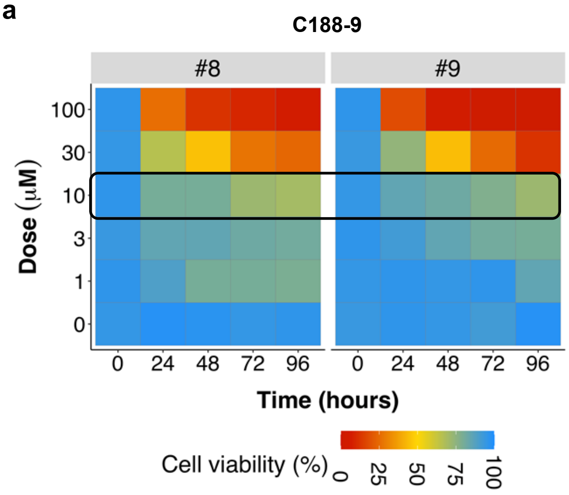
C188-9
#8
#9
100
30
10
3
1
0
Dose (μM)
0 24 48 72 96
0 24 48 72 96
Time (hours)
Cell viability (%)
0
25
50
75
100

Supplementary Fig. 5. STAT-3 is activated in the proximity of VAT in CRC patients with obesity

a, Heatmap matrix of CMS2 CR-CSphC (CSphC #8, 9) viability following treatment with dose-escalation of STAT3 inhibitor (C188-9), at the indicated time points. Black boxes indicate the selected concentration for *in vitro* studies.

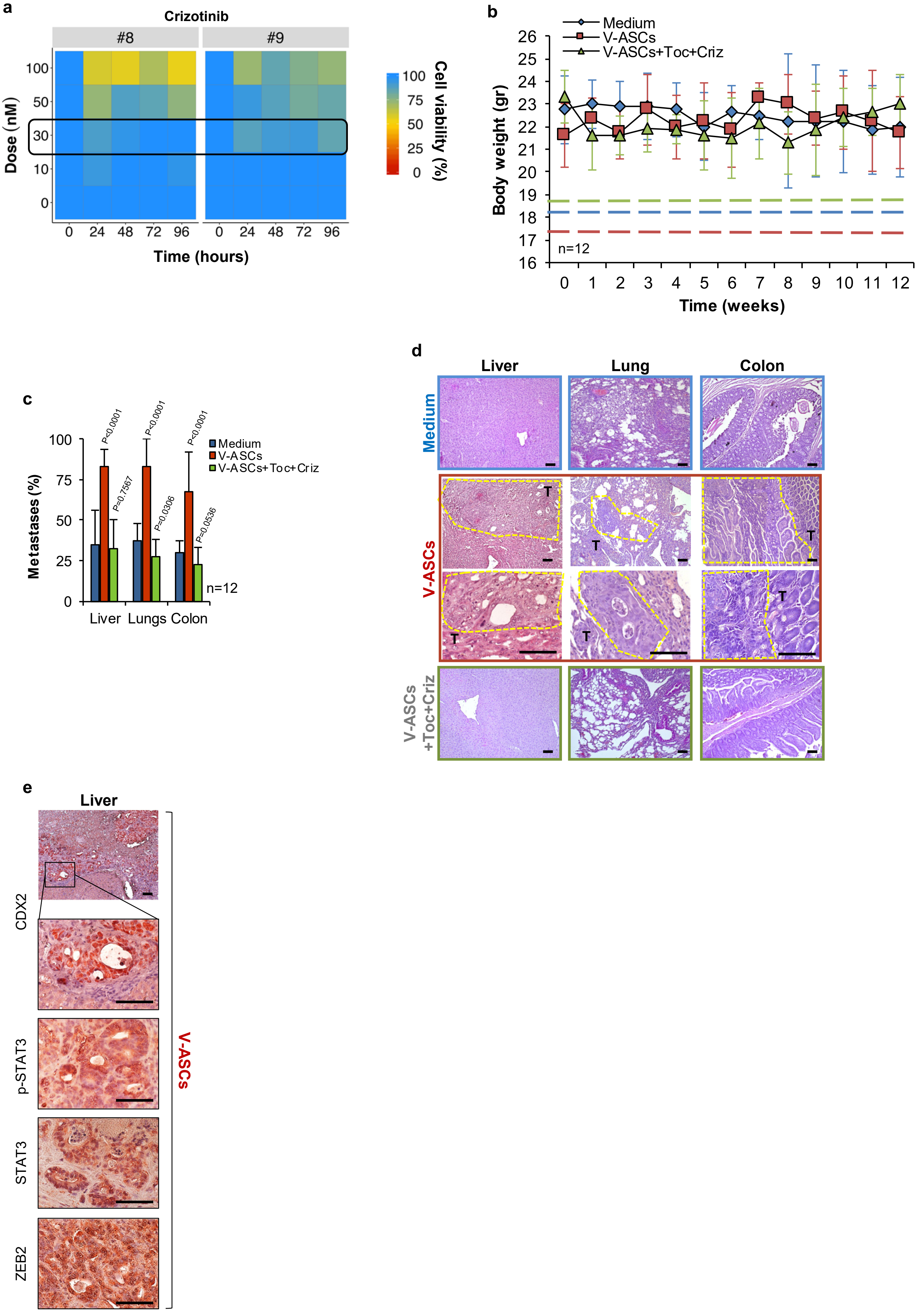

Di Franco et al. Supplementary Figure 6

Supplementary Fig. 6. IL-6R and c-Met blockade prevents the metastasis formation of CR-CSphCs induced by VAT.

a, Heatmap matrix of CMS2 CR-CSphC (CSphC #8, 9) viability following treatment with dose-escalation of crizotinib, at the indicated time points. Black boxes indicate the selected concentration for *in vitro* studies. **b**, Kinetics of body weight variation in mice following intrasplenic injection of LUC-GFP CMS2 CR-CSphCs (#8, 9) alone (Medium) or co-injected with V-ASCs, at the indicated time points. Mice were treated i.p. with tocilizumab (Toc) and crizotinib (Criz). Dotted lines represent the maximum tolerated reduction in body weight, measured as 80% of basal weight of mice belonging to each treatment group. Data are mean of 12 independent experiments in mice treated as indicated. **c**, Metastasis formation in mice treated as indicated, 9 weeks after treatment suspension. **d**, H&E analysis of liver, lung and colon metastatic lesions derived from mice treated as indicated. Scale bars, 200 μm. T: tumor. **e**, Representative immunohistochemical analysis of CDX2, p-STAT3, STAT3, and ZEB2 in paraffin-embedded sections of tumor xenograft specimens of CRC liver metastasis in mice intrasplenically co-injected with CR-CSphCs and V-ASCs. Scale bars, 100 μm. For (d-e) one representative of 12 independent experiments is shown. Statistical significance between 2 groups was determined by unpaired Student's t-test (2-tailed).

Supplementary Table 1. Human primary ASC cell lines

ASC #	2	3	4	5	6	7	8	9	10	11	12	13	14	16	17	18	20	21
Subcutaneous								N/A	N/A	N/A	N/A					N/A		
Visceral	N/A		N/A			N/A						N/A		N/A	N/A		N/A	N/A
BMI	43	25	25	28	24	25	21	26	24	61	N/A	21	30	N/A	30	20	23	24

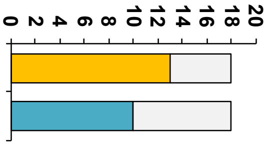

Supplementary Table 2. Mutational, MSI and CMS profile of colorectal cancer sphere cell lines (CR-CSphC).

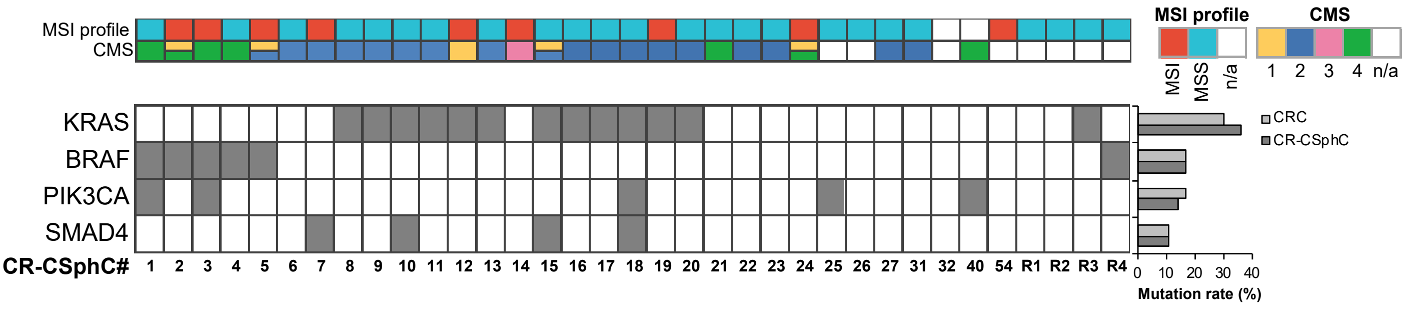

Supplementary Table 3. Primer sequences and source.

Gene Symbol	Primer sequence/Catalog #	Source
NGF	Cat. # PPH00205F	Qiagen
BDNF	Cat. # PPH00569F	Qiagen
NTF3	Cat. # PPH00687A	Qiagen
NTF4	Cat. # PPH01123A	Qiagen
ZEB1	Cat. # PPH01922A	Qiagen
ZEB2	Cat. # PPH09021B	Qiagen
CDX2	Fwd: 5-TTCACTACAGTCGCTACATCACC-3<br><br>Rev: 5-TTGTTGATTTTCCTCTCCTTTGC-3	Metabion
E-CADHERIN	Fwd: 5-TGGAGGAATTCTTGCTTTGC-3<br><br>Rev: 5-CGCTCTCCTCCGAAGAAAC-3	Metabion
CXCR4	Fwd: 5-AGCATGACGGACAAGTACAGG-3<br><br>Rev: 5-GATGAAGTCGGGAATAGTCAGC-3	Metabion
FRMD6	Fwd: 5-GGACACTCTGGGTTGATTGTG-3<br><br>Rev: 5-TGTGTCGATCAGTGGAGGTC-3	Metabion
N-CADHERIN	Fwd: 5-ACAGTGGCCACCTACAAAGG-3<br><br>Rev: 5-CCGAGATGGGGTTGATAATG-3	Metabion
SLUG	Fwd: 5-GGTCAAGAAGCATTTCAACG-3<br><br>Rev: 5-CACAGTGATGGGGCTGTATG-3	Metabion
SNAIL	Fwd: 5-GGGAATTCTATGCCGCGCTCTTTCCTCGTC-3<br><br>Rev: 5-GGGGATCCTCAGCGGGGACATCCTGAGCAG-3	Metabion
TWIST	Fwd: 5-GGCATCACTATGGACTTTCTCTATT-3<br><br>Rev: 5-GGCCAGTTTGATCCCAGTATT-3	Metabion
VIMENTIN	Fwd: 5-TTCCTGGGCTACGACCATAC-3<br><br>Rev: 5-TGTGCTCCATCAAGCAATTC-3	Metabion
GAPDH	Fwd: 5-GCT TCG CTC TCT GCT CCT CCT GT-3<br><br>Rev: 5-TAC GAC CAA ATC CGT TGA CTC CG-3	Metabion
