# Supplementary material for: Adipose stem cell niche reprograms the colorectal cancer stem cell metastatic machinery
Source: Nat Commun. 2021 Aug 18;12:5006. doi: 10.1038/s41467-021-25333-9 (PMC8373975; doi:10.1038/s41467-021-25333-9)
Supplement: Supplementary file 3 — Reporting summary [file 41467_2021_25333_MOESM3_ESM.pdf]

nature research

Corresponding author(s): Giorgio Stassi, M.D.

Last updated by author(s): May 7, 2021

Reporting Summary

Nature Research wishes to improve the reproducibility of the work that we publish. This form provides structure for consistency and transparency in reporting. For further information on Nature Research policies, see our Editorial Policies and the Editorial Policy Checklist.

Statistics

For all statistical analyses, confirm that the following items are present in the figure legend, table legend, main text, or Methods section.

n/a	Confirmed	
☐	☒	The exact sample size (*n*) for each experimental group/condition, given as a discrete number and unit of measurement
☐	☒	A statement on whether measurements were taken from distinct samples or whether the same sample was measured repeatedly
☐	☒	The statistical test(s) used AND whether they are one- or two-sided<br>*Only common tests should be described solely by name; describe more complex techniques in the Methods section.*
☐	☒	A description of all covariates tested
☐	☒	A description of any assumptions or corrections, such as tests of normality and adjustment for multiple comparisons
☐	☒	A full description of the statistical parameters including central tendency (e.g. means) or other basic estimates (e.g. regression coefficient) AND variation (e.g. standard deviation) or associated estimates of uncertainty (e.g. confidence intervals)
☐	☒	For null hypothesis testing, the test statistic (e.g. *F*, *t*, *r*) with confidence intervals, effect sizes, degrees of freedom and *P* value noted<br>*Give P values as exact values whenever suitable.*
☒	☐	For Bayesian analysis, information on the choice of priors and Markov chain Monte Carlo settings
☒	☐	For hierarchical and complex designs, identification of the appropriate level for tests and full reporting of outcomes
☒	☐	Estimates of effect sizes (e.g. Cohen's *d*, Pearson's *r*), indicating how they were calculated

Our web collection on statistics for biologists contains articles on many of the points above.

Software and code

Policy information about availability of computer code

Data collection	*Provide a description of all commercial, open source and custom code used to collect the data in this study, specifying the version used OR state that no software was used.*
Data analysis	*Provide a description of all commercial, open source and custom code used to analyse the data in this study, specifying the version used OR state that no software was used.*

For manuscripts utilizing custom algorithms or software that are central to the research but not yet described in published literature, software must be made available to editors and reviewers. We strongly encourage code deposition in a community repository (e.g. GitHub). See the Nature Research guidelines for submitting code & software for further information.

Data

Policy information about availability of data

All manuscripts must include a data availability statement. This statement should provide the following information, where applicable:

- Accession codes, unique identifiers, or web links for publicly available datasets
- A list of figures that have associated raw data
- A description of any restrictions on data availability

The data that support the findings of this study are available from the corresponding author, GS, upon request.

April 2020

Field-specific reporting

Please select the one below that is the best fit for your research. If you are not sure, read the appropriate sections before making your selection.

☒ Life sciences ☐ Behavioural & social sciences ☐ Ecological, evolutionary & environmental sciences

For a reference copy of the document with all sections, see nature.com/documents/nr-reporting-summary-flat.pdf

Life sciences study design

All studies must disclose on these points even when the disclosure is negative.

Sample size	No sample-size calculation was performed.
Data exclusions	No data were excluded from the analyses.
Replication	All the attempts at replication were successful.
Randomization	Mice were randomly divided into the experimental groups reported in the manuscript.
Blinding	For the CRC patients' survival analysis, consensus molecular subtype allocation of primary cell lines, RNAseq, miRNA analysis, and in vivo model experiments (primary and metastatic potential assessment), investigators were blinded to group allocation during data collection and analysis.

Reporting for specific materials, systems and methods

We require information from authors about some types of materials, experimental systems and methods used in many studies. Here, indicate whether each material, system or method listed is relevant to your study. If you are not sure if a list item applies to your research, read the appropriate section before selecting a response.

Materials & experimental systems

n/a	Involved in the study	
☐	☒	Antibodies
☐	☒	Eukaryotic cell lines
☒	☐	Palaeontology and archaeology
☐	☒	Animals and other organisms
☒	☐	Human research participants
☐	☒	Clinical data
☒	☐	Dual use research of concern

Methods

n/a	Involved in the study	
☒	☐	ChIP-seq
☐	☒	Flow cytometry
☒	☐	MRI-based neuroimaging

Antibodies

Antibodies used	Information about all the antibodies used in the study has been reported in the "Methods" section of the manuscript, including supplier and clone name.
Validation	The antibodies used in our study were validated by the manufacturer.

Eukaryotic cell lines

Policy information about cell lines

Cell line source(s)	The protocol for isolation and propagation of primary cell lines, from colorectal cancer and adipose tissue, has been described in the "Methods" section of the manuscript.
Authentication	As reported in the manuscript, "CR-CSphC lines were routinely authenticated by short tandem repeat (STR) analysis using a multiplex PCR assay including a set of 24 loci (GlobalFiler™ STR kit, Applied Biosystem), by comparing them to the parental patient tumor tissues."
Mycoplasma contamination	All cell lines used in the study have been previously tested for mycoplasma contamination using the MycoAlertTM Mycoplasma Detection Kit (Lonza, LT07-318).
Commonly misidentified lines (See ICLAC register)	*Name any commonly misidentified cell lines used in the study and provide a rationale for their use.*

Animals and other organisms

Policy information about studies involving animals; ARRIVE guidelines recommended for reporting animal research

Laboratory animals	As reported in the manuscript, "Six weeks old male NOD-SCID mice were purchased by Charles River Laboratories"
Wild animals	*Provide details on animals observed in or captured in the field; report species, sex and age where possible. Describe how animals were caught and transported and what happened to captive animals after the study (if killed, explain why and describe method; if released, say where and when) OR state that the study did not involve wild animals.*
Field-collected samples	As reported in the manuscript, "Mice were maintained in a temperature controlled system with a 12 hours dark/light cycle, with ad libitum access to pelleted chow (Special Diets Services-811900 VRF1 (P)) and to 0.45 µm filtered water in sterile drinking bottles, in cages (Tecniplast) with radiation-sterilized bedding (SAWI Research Bedding, JELU-WERK)."
Ethics oversight	As reported in the manuscript, "Animal were housed at the University of Palermo in accordance to institutional guidelines of the Italian animal welfare (D.L. n° 26 March 4, 2014) and authorization n. 951/2015-PR."

Note that full information on the approval of the study protocol must also be provided in the manuscript.

Clinical data

Policy information about clinical studies

All manuscripts should comply with the ICMJEguidelines for publication of clinical research and a completedCONSORT checklist must be included with all submissions.

Clinical trial registration	*Provide the trial registration number from ClinicalTrials.gov or an equivalent agency.*
Study protocol	*Note where the full trial protocol can be accessed OR if not available, explain why.*
Data collection	*Describe the settings and locales of data collection, noting the time periods of recruitment and data collection.*
Outcomes	*Describe how you pre-defined primary and secondary outcome measures and how you assessed these measures.*

Flow Cytometry

Plots

Confirm that:

- [x] The axis labels state the marker and fluorochrome used (e.g. CD4-FITC).
- [x] The axis scales are clearly visible. Include numbers along axes only for bottom left plot of group (a 'group' is an analysis of identical markers).
- [ ] All plots are contour plots with outliers or pseudocolor plots.
- [x] A numerical value for number of cells or percentage (with statistics) is provided.

Methodology

Sample preparation	As described in the manuscript, "For flow cytometry analysis, cells were collected, washed in PBS and stained for 1 hour at 4 ° C with specific conjugated antibodies or corresponding IMC. Dead cells' exclusion was performed by using 7-AAD (0.25 µg/1x106 cells, BD Biosciences). Before analysis, cells were washed with 2% BSA and 2 mM EDTA PBS and filtered with a 70 µM mesh to prevent cell clogging."
Instrument	BD FACSLyric and BD FACSMelody have been used for flow cytometry analysis and cell sorting, respectively.
Software	Flow cytometry data have been analyzed using FlowJo v10 software.
Cell population abundance	As reported in the manuscript, "Postsorting analysis was performed to verify the purity of sorted population.". The purity of sorted cell population is included in the Supplementary Information file.
Gating strategy	Before flow cytometry analysis, or cell sorting, a gating strategy has been performed in order to exclude cell debris (SSC-A versus FSC-A), to exclude doublets and clumps (FSC-H versus FSC-A), and to exclude dead cells (7-AAD versus FSC-A).

- [x] Tick this box to confirm that a figure exemplifying the gating strategy is provided in the Supplementary Information.
