# Supplementary material for: Adipose stem cell niche reprograms the colorectal cancer stem cell metastatic machinery
Source: Nat Commun. 2021 Aug 18;12:5006. doi: 10.1038/s41467-021-25333-9 (PMC8373975; doi:10.1038/s41467-021-25333-9)
Supplement: Supplementary file 4 — Source Data [file 41467_2021_25333_MOESM4_ESM.zip › Di Franco et al Source Data file NCOMMS-20-35780B.pptx]

## Slide 1
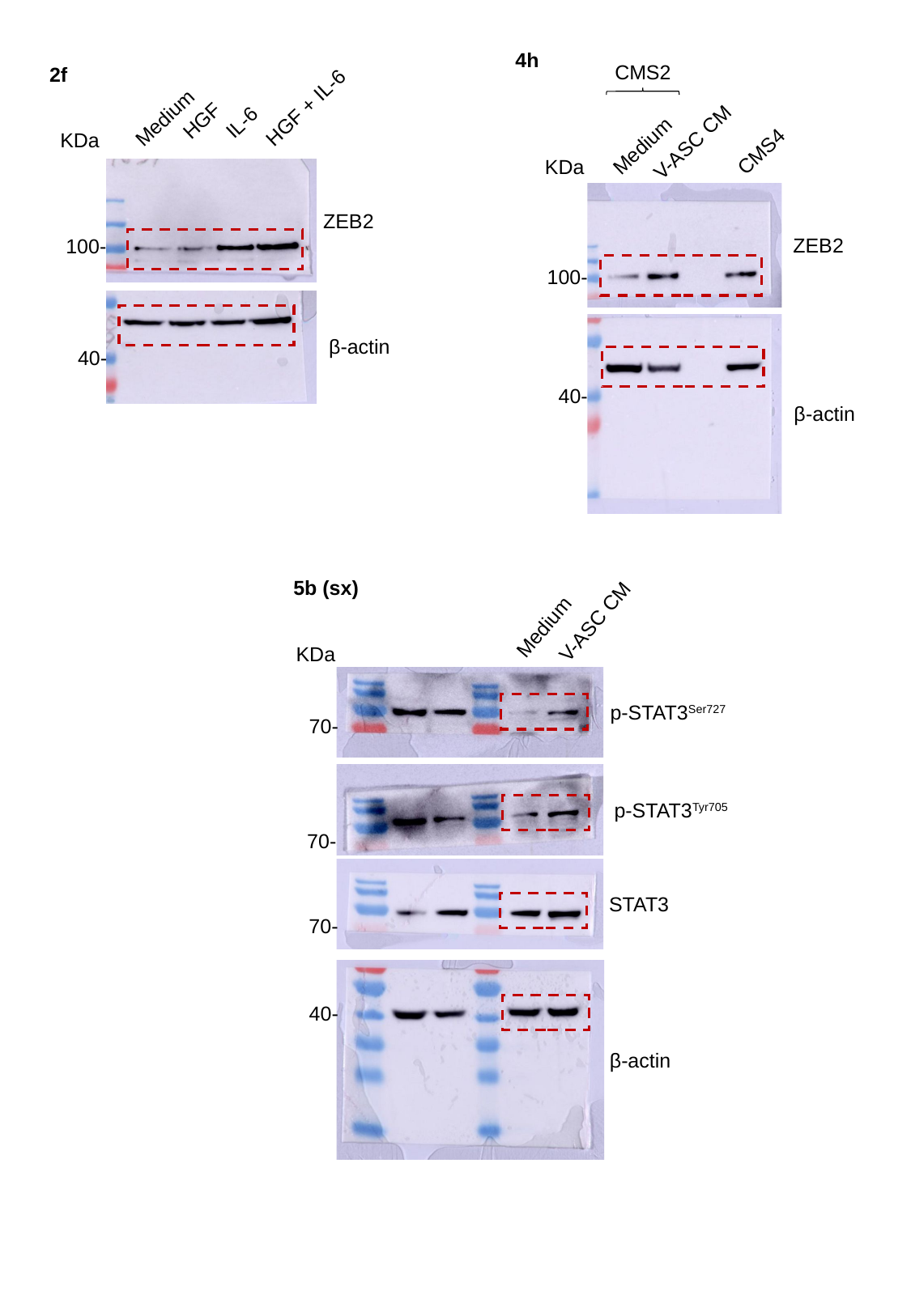

4h
CMS2
V-ASC CM
Medium
CMS4
KDa
ZEB2
100-
40-
β-actin
2f
HGF + IL-6
Medium
HGF
IL-6
KDa
ZEB2
100-
β-actin
40-
5b (sx)
V-ASC CM
Medium
KDa
p-STAT3Ser727
70-
p-STAT3Tyr705
70-
STAT3
70-
40-
β-actin

## Slide 2
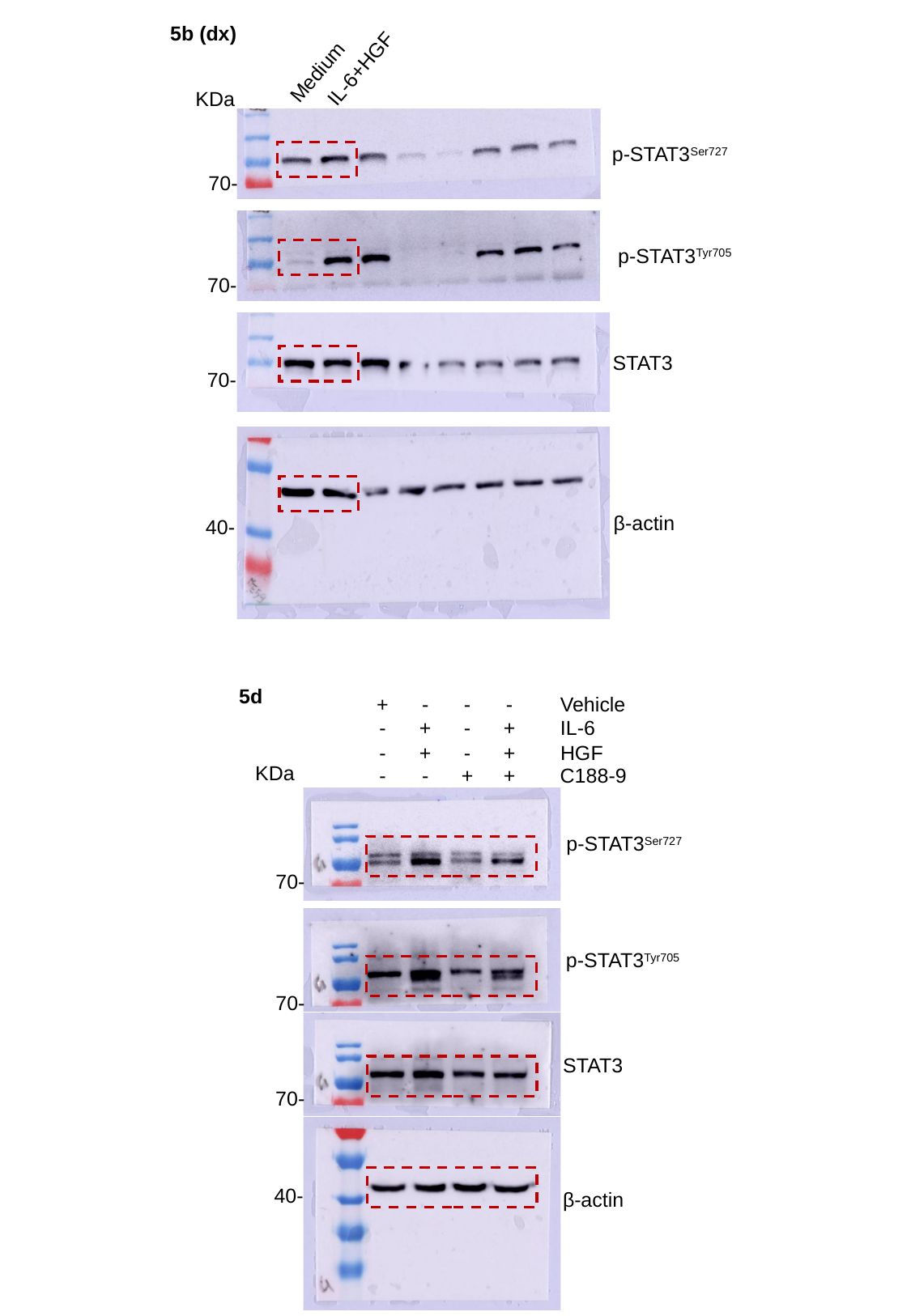

5b (dx)
IL-6+HGF
Medium
KDa
p-STAT3Ser727
70-
p-STAT3Tyr705
70-
STAT3
70-
β-actin
40-
5d
+
-
-
-
Vehicle
-
+
-
+
IL-6
-
+
-
+
HGF
KDa
-
-
+
+
C188-9
p-STAT3Ser727
70-
p-STAT3Tyr705
70-
STAT3
70-
40-
β-actin

## Slide 3
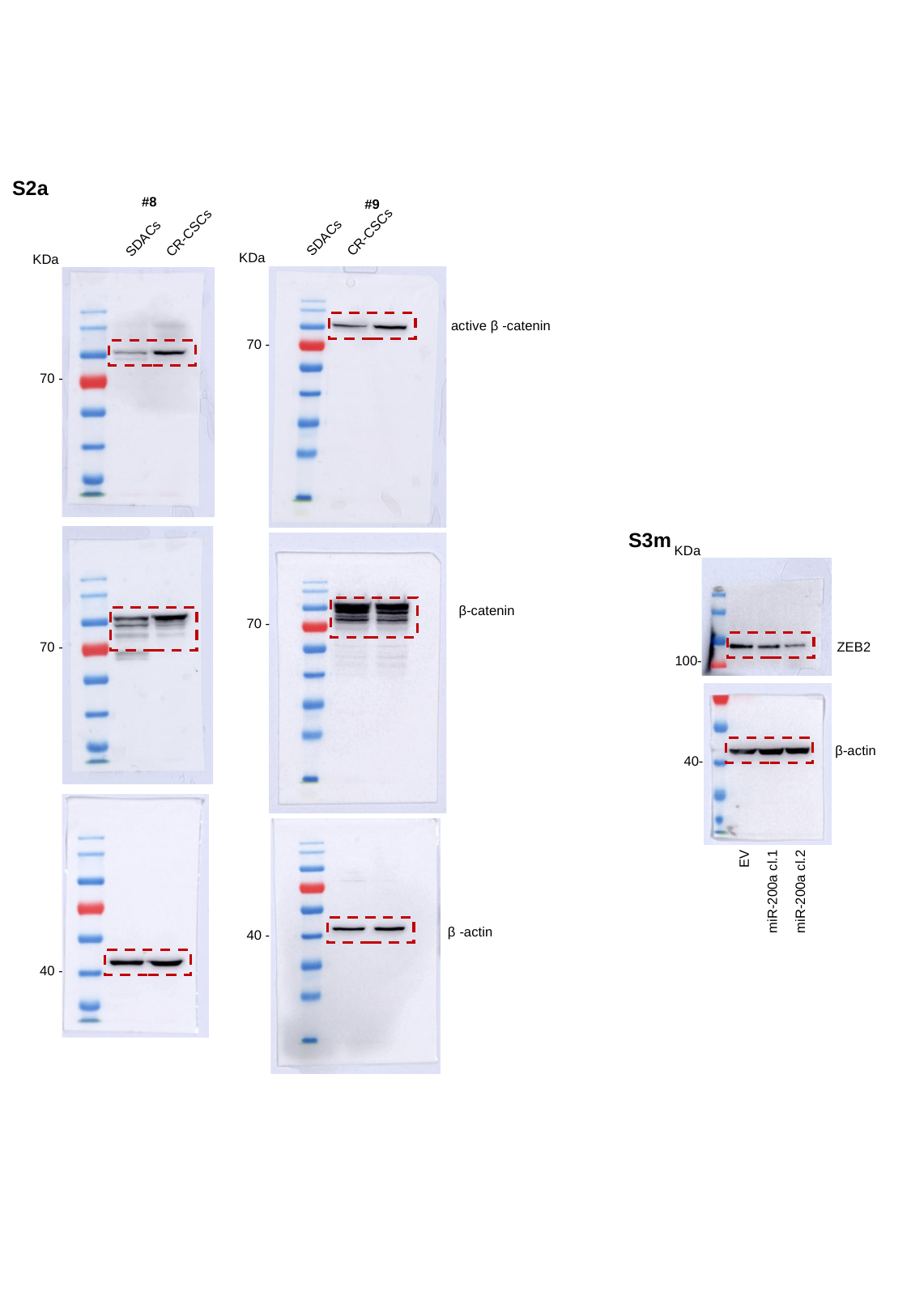

S2a
#8
#9
CR-CSCs
CR-CSCs
SDACs
SDACs
KDa
KDa
active β -catenin
70 -
70 -
β-catenin
70 -
70 -
β -actin
40 -
40 -
S3m
KDa
ZEB2
100-
β-actin
40-
EV
miR-200a cl.1
miR-200a cl.2

## Slide 4
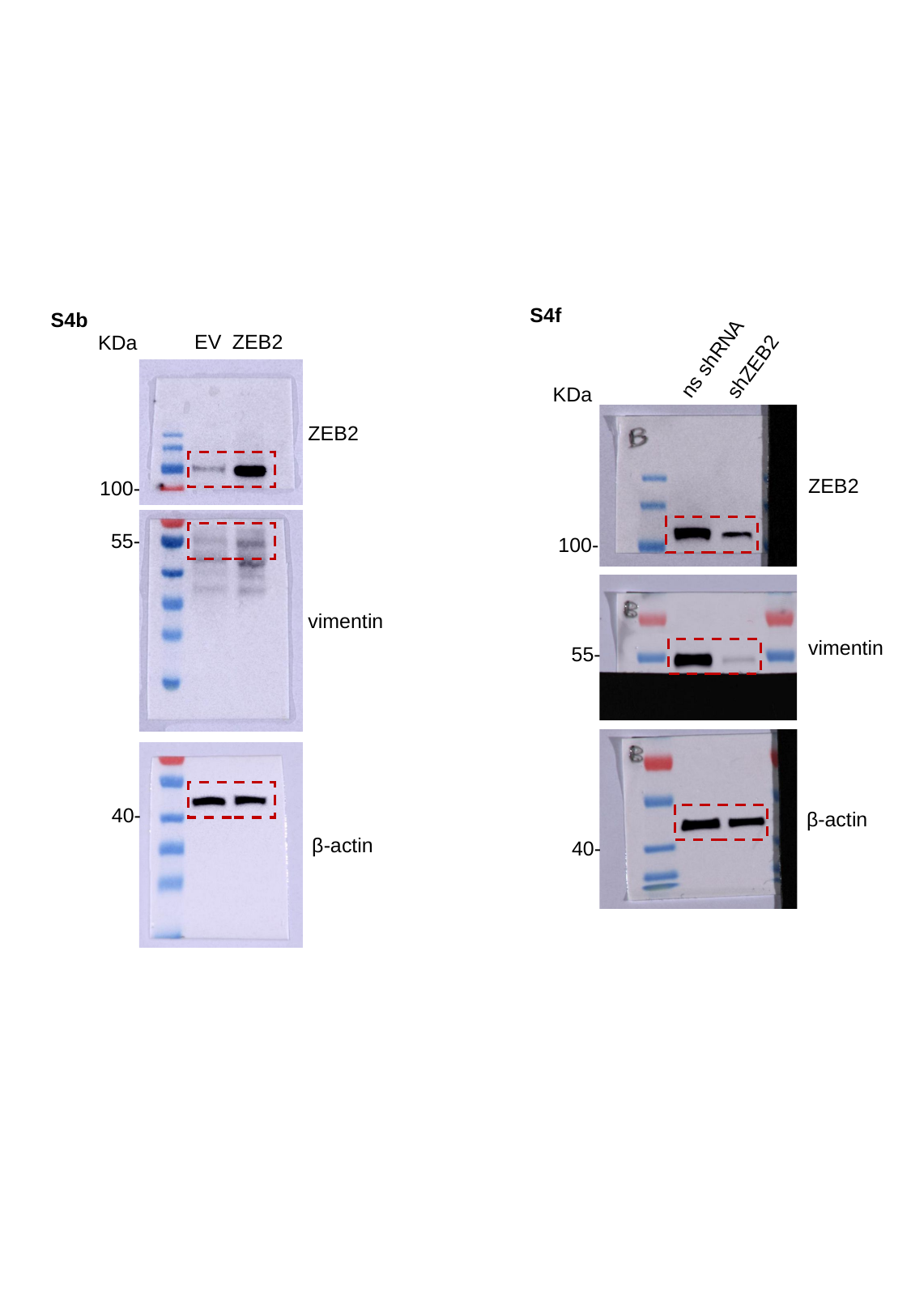

S4f
ns shRNA
shZEB2
KDa
ZEB2
100-
vimentin
55-
β-actin
40-
S4b
EV
ZEB2
KDa
ZEB2
100-
55-
vimentin
40-
β-actin
